# Long-term anti-inflammatory diet in relation to improved breast cancer prognosis: a prospective cohort study

**DOI:** 10.1038/s41523-020-00179-4

**Published:** 2020-08-13

**Authors:** Kang Wang, Jia-Zheng Sun, Qian-Xue Wu, Zhu-Yue Li, Da-Xue Li, Yong-Fu Xiong, Guo-Chao Zhong, Yang Shi, Qing Li, Jiali Zheng, Nitin Shivappa, James R. Hébert, Theodoros Foukakis, Xiang Zhang, Hong-Yuan Li, Ting-Xiu Xiang, Guo-Sheng Ren

**Affiliations:** 1 Department of the Endocrine and Breast Surgery, The First Affiliated hospital of Chongqing Medical University, Chongqing Medical University, Chongqing, 400016 China; 2 Key Laboratory of Molecular Oncology and Epigenetics, The First Affiliated Hospital of Chongqing Medical University, Chongqing, China; 3 West China Hospital/West China School of Nursing, Sichuan University, Chengdu, China; 4 Department of Breast Surgery, Chongqing Health Center for Women and Children, Chongqing, 400000 China; 5 The First Department of Hepatobiliary Surgery, Affiliated Hospital of North Sichuan Medical College, Nanchong, 637007 China; 6 Department of Hepatobiliary Surgery, The Second Affiliated Hospital of Chongqing Medical University, Chongqing, China; 7 Division of Biostatistics and Data Science, Department of Population Health Sciences, Medical College of Georgia, Augusta University, Augusta, GA USA; 8 Department of Epidemiology and Biostatistics, Arnold School of Public Health, University of South Carolina, Columbia, SC 29208 USA; 9 Cancer Prevention and Control Program, University of South Carolina, Columbia, SC 29208 USA; 10 Connecting Health Innovations, LLC, Columbia, SC 29201 USA; 11 Department of Oncology-Pathology, Karolinska Institutet, 17164 Stockholm, Sweden; 12 Breast Center, Theme Cancer, Karolinska University Hospital, 17176 Stockholm, Sweden

**Keywords:** Risk factors, Breast cancer

## Abstract

Inflammation-modulating nutrients and inflammatory markers are established cancer risk factors, however, evidence regarding the association between post-diagnosis diet-associated inflammation and breast cancer survival is relatively sparse. We aimed to examine the association between post-diagnosis dietary inflammatory index (DII®) and risks of all-cause and breast cancer-specific mortality. A total of 1064 female breast cancer survivors in the Prostate, Lung, Colorectal, and Ovarian Cancer Screening (PLCO) Trial prospective cohort, were included in this analysis if they had completed the diet history questionnaire (DHQ). Energy-adjusted DII (E-DII^TM^) scores were calculated based on food and supplement intake. Cox regression and competing risk models were used to estimate multivariable-adjusted hazards ratios (HRs) and 95% confidence intervals (95% CIs) by E-DII tertile (T) for all-cause and breast cancer-specific mortality. With median follow-up of 14.6 years, there were 296 (27.8%) deaths from all causes and 100 (9.4%) breast cancer-specific death. The E-DII was associated with all-cause mortality (HR T3 vs T1, 1.34; 95% CI, 1.01–1.81; *P*_trend_, 0.049, Table 2) and breast cancer mortality (HR T3 vs T1, 1.47; 95% CI, 0.89–2.43; *P*_trend_, 0.13; multivariable-adjusted HR for 1-unit increment: 1.10; 95% CI: 1.00–1.22). Non-linear positive dose–response associations with mortality from all causes were identified for E-DII scores (*P*_non-linearity_ < 0.05). The post-diagnosis E-DII was statistically significantly associated with mortality risk among breast cancer survivors. Long-term anti-inflammatory diet might be a means of improving survival of breast cancer survivors.

## Introduction

Approximately 268,600 women were diagnosed with invasive breast cancer in the United States in 2019^1^. Breast cancer is rapidly becoming more common in other parts of the world^2^. In the past 40 years, breast cancer screening and treatments have improved substantially. Previous studies suggest that screening for early detection and improved treatment, including adjuvant therapy, have contributed to about one-half of the decline in breast cancer-related mortality^3^. Mortality can be lowered yet more by applying comprehensive disease management^4^. The importance of healthful diets for survivorship had been indicated in several studies^5,6^. However, debate continues about the best strategies for the dietary management of breast cancer patients to improve long-term survival^7^.

Chronic inflammation is implicated in breast cancer, and studies suggest a link between inflammation and breast cancer outcomes^8–11^. More recently, literature has emerged that offers contradictory findings regarding the relationship between survival after a breast cancer diagnosis and specific nutrients known to modulate inflammation, such as dietary fat, fruits, vegetables, fiber, and alcohol^12–15^. The overall inflammatory potential of the diet may provide better insights into the effect of diet on breast cancer survival than assessing only a single nutrient; after all, a typical human diet consists of a variety of both proinflammatory and anti-inflammatory foods and nutrients^16^. The dietary inflammatory index (DII®) reflects both a robust literature base and standardization of individual intakes to global referent values^17^. To date, a prospective cohort study from the Women’s Health Initiative Study has used the DII to assess the association between post-diagnosis dietary inflammatory potential and survival of invasive breast cancer patients, indicating that consuming a more anti-inflammatory diet after breast cancer diagnosis reduced risk of death from cardiovascular disease^18^. Two retrospective cohort studies found inconsistent results: Jang et al. showed that anti-inflammatory diets may decrease the risk of cancer recurrence and overall mortality in patients with breast cancer^19^, while findings from Italy did not suggest an association between the inflammatory potential of diet and the survival of female breast cancer patients^20^. Retrospective studies are susceptible to both selection and information biases. For example, it has been noted that positive dietary changes were more common among younger women or those who underwent chemotherapy^21^, and it is commonly assumed that dietary data are susceptible to biases associated with known outcomes, as in case-control studies^22,23^. Furthermore, some clinicopathological or lifestyle factors may mediate the association of diet and disease risk or survival, such as age^24^, hormone receptor status^25^, body mass index (BMI)^5,26^, gene polymorphisms^27^, and smoking status^28^.

The energy-adjusted DII (E-DII^TM^) is a composite measure of diet-associated inflammation which takes into account inflammatory effect of overall diet of an individual as opposed to looking at specific nutrients or food items. In this study, we aimed to comprehensively assess the association between inflammatory potential of post-diagnosis diet, as estimated by the E-DII, and all-cause and breast cancer-specific mortality among post-menopausal breast cancer patients from the Prostate, Lung, Colorectal, and Ovarian Cancer Screening Trial (PLCO) prospective cohort.

## Results

### Patients characteristics

**Fig. 1:**
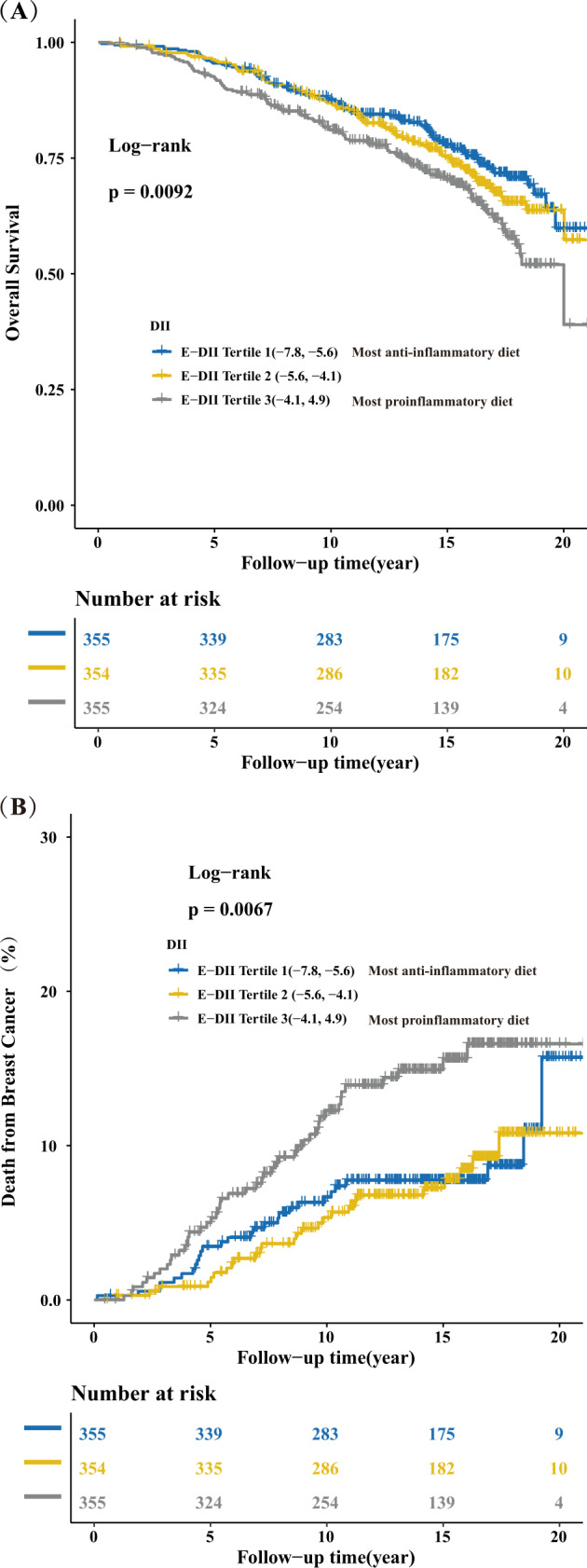
Survival analysis between E-DII scores in T1, T2, and T3. Kaplan–Meier curves of **a** overall survival; **b** breast cancer-specific mortality.

After a median follow-up time of 14.6 years from breast cancer diagnosis (interquartile range, 10.5–16.8 years), a total of 1064 eligible female breast cancer cases were available for analyses (Supplementary Fig. 1). Among them, there were 296 (27.8%) all-cause deaths, and 100 (9.4%) breast cancer-specific deaths. The 10-year overall survival rates were 87.3%, 86.6%, and 80.7% in tertile (T) 1, T2, and T3 group, respectively (log-rank test, *P* < 0.001; HR, 1.16; 95% CI, 0.87–1.55 (T2 vs. T1), HR, 1.53; 95% CI, 1.15–2.03 (T3 vs. T1), Table 2 and Fig. 1a). Similarly, the 10-year breast cancer-specific survival rates were 93.2%, 94.5, and 88.1% in T1, T2, and T3 group, respectively (log-rank test, *P* < 0.001; HR, 1.76; 95% CI, 1.10–2.82 (T2 vs. T1), HR, 1.70; 95% CI, 1.17–2.47 (T3 vs. T1), Table 2 and Fig. 1b). As shown in Table 1, compared with cases with the most anti-inflammatory diets (i.e., −7.8 to −5.6, E-DII T1), patients consuming the most pro-inflammatory diets (i.e., −4.1 to 4.9, E-DII T3) had a higher total energy intake, higher BMI, more current hormone therapy, and had lower education level, shorter follow-up time and time from breast cancer diagnosis to DHQ completion, and less aspirin use. The baseline characteristics of participants by tertile of E-DII from food, but without supplement, were shown as Supplementary Table 1.

**Table 1:** Baseline characteristics of 1064 breast cancer cases in the PLCO Cancer Screening Trial by tertile of E-DII from food plus supplement.

	Post-diagnostic exposure to dietary inflammatory potential			
	E-DII tertile 1 (−7.8, −5.6)	E-DII tertile 2 (−5.6, −4.1)	E-DII tertile 3 (−4.1, 4.9)	*P*
Number of cases	355	354	355	
	Median (IQR)	Median (IQR)	Median (IQR)	
Age at breast cancer diagnosis (years)	65 (61, 70)	66 (62, 71)	65 (60.5, 69.5)	0.26^a^
Total energy intake (kcal/day)	1398.7 (1084.2, 1743.2)	1410.6 (1110.4, 1792.7)	1526.6 (1189.2, 1905.8)	0.009^a^
Alcohol intake (g/day)	1.0 (0, 5.9)	0.87 (0, 4.6)	0.87 (0, 5.0)	0.59^a^
Years from breast cancer diagnosis to DHQ completion (years)	1.4 (0.4, 2.3)	1.4 (0.4, 2.4)	1.1 (0.1, 2.1)	0.01^a^
Person-years of follow-up since breast cancer diagnosis	15.0 (11.0, 17.3)	15.2 (11.3, 16.9)	13.7 (9.4, 16.3)	<0.001^a^
	*N* (%)^b^	*N* (%)^b^	*N* (%)^b^	
Trial arm				
Intervention	187 (52.7)	187 (52.8)	193 (54.4)	0.88^b^
Control	168 (47.3)	167 (47.2)	162 (45.6)	
Race/Ethnicity				
White	315 (88.7)	326 (92.1)	329 (92.7)	0.005^c^
Black	8 (2.3)	10 (2.8)	15 (4.2)	
Hispanic	1 (0.3)	4 (1.1)	2 (0.6)	
Asian	28 (7.9)	12 (3.4)		
Other^d^	3 (0.8)	2 (0.6)	3 (0.8)	
BMI (kg/m^2^)				
≤18.5	1 (0.3)	3 (0.8)	3 (0.8)	<0.001^c^
18.6–25	175 (49.3)	167 (47.2)	124 (34.9)	
26–30	119 (33.5)	114 (32.2)	125 (35.2)	
31–50	60 (16.9)	70 (19.8)	103 (29.0)	
Marital status				
Single^e^	18 (5.1)	11 (3.1)	15 (4.2)	0.83^b^
Married or living as married	252 (71.0)	260 (73.4)	247 (69.6)	
Divorced or separated	38 (10.7)	39 (11.0)	41 (11.5)	
Widowed	47 (13.2)	44 (12.4)	52 (14.6)	
Education level				
Less than high school	13 (3.7)	16 (4.5)	33 (9.3)	<0.001^b^
High school graduate or equivalent	62 (17.5)	78 (22.0)	98 (27.6)	
Post-high school education	34 (9.6)	44 (12.4)	37 (10.4)	
College education or higher	246 (69.3)	216 (61.0)	187 (52.7)	
Income level				
<$20,000	52 (14.6)	55 (15.5)	63 (17.7)	0.16^c^
$20,000-$49,000	149 (42.0)	143 (40.4)	171 (48.2)	
$50,000-$99,000	123 (34.6)	131 (37.0)	94 (26.5)	
$100,000-$200,000	28 (7.9)	21 (5.9)	23 (6.5)	
<$200,000	3 (0.8)	4 (1.1)	4 (1.1)	
Smoking status				
Never smoked	181 (51.0)	196 (55.4)	187 (52.7)	0.005^b^
Past smoker	16 (4.5)	26 (7.3)	40 (11.3)	
Current smoker	158 (44.5)	132 (37.3)	128 (36.1)	
Physical activity				
Active less than one time per month	48 (13.5)	40 (11.3)	52 (14.6)	0.41^b^
Active at least one time per month	307 (86.5)	314 (88.7)	303 (85.4)	
Hormone therapy				
Never used	112 (31.5)	109 (30.8)	139 (39.2)	0.03^b^
Former used	226 (63.7)	235 (66.4)	198 (55.8)	
Current used	17 (4.8)	10 (2.8)	18 (5.1)	
Birth control pills				
No	151 (42.5)	171 (48.3)	167 (47)	0.27
Yes	204 (57.5)	183 (51.7)	188 (53.0)	
Aspirin use				
None	99 (27.9)	77 (21.8)	110 (31.0)	0.01^b^
<Once/week	74 (20.8)	81 (22.9)	91 (25.6)	
Once per week or more	182 (51.3)	196 (55.4)	154 (43.4)	
Number of live babies delivered				
0	37 (10.4)	32 (9.0)	45 (12.7)	0.1^b^
1–2	138 (38.9)	124 (35.0)	107 (30.1)	
≥3	180 (50.7)	198 (55.9)	203 (57.2)	
Breast feeding				
None or never pregnant	137 (38.6)	146 (41.2)	176 (49.6)	0.06^b^
<6 months	101 (28.5)	91 (25.7)	91 (25.6)	
6–11 months	64 (18.0)	60 (16.9)	43 (12.1)	
>12 months	53 (14.9)	57 (16.1)	45 (12.7)	
Oophorectomy status				
Ovaries not removed	298 (83.9)	278 (78.5)	285 (80.3)	0.17^b^
removed	57 (16.1)	76 (21.5)	70 (19.7)	
Family history of breast cancer				
No	288 (81.1)	275 (77.7)	287 (80.8)	0.17^c^
Yes	63 (17.7)	79 (22.3)	66 (18.6)	
Possible	4 (1.1)	0 (0.0)	2 (0.6)	
History of diabetes				
No	331 (93.2)	333 (94.1)	326 (91.8)	0.50^b^
Yes	24 (6.8)	21 (5.9)	29 (8.2)	
Stage				
In situ	68 (19.2)	78 (22.0)	70 (19.7)	0.27^c^
I	193 (54.4)	172 (48.6)	170 (47.9)	
II	86 (24.2)	97 (27.4)	100 (28.2)	
III	8 (2.3)	7 (2.0)	15 (4.2)	
Nuclear grade				
I	107 (30.1)	102 (28.8)	108 (30.4)	0.98^b^
II	150 (42.3)	156 (44.1)	148 (41.7)	
III	98 (27.6)	96 (27.1)	99 (27.9)	
ER status				
Positive	308 (86.8)	300 (84.7)	292 (82.3)	0.25^b^
Negative	47 (13.2)	54 (15.3)	63 (17.7)	
PR status				
Positive	268 (75.5)	263 (74.3)	269 (75.8)	0.89^b^
Negative	87 (24.5)	91 (25.7)	86 (24.2)	
Surgery				
Lumpectomy	153 (43.1)	153 (43.2)	153 (43.1)	1.00^b^
Mastectomy	157 (44.2)	157 (44.4)	158 (44.5)	
Others^f^	45 (12.7)	44 (12.4)	44 (12.4)	

*E-DII* energy-adjusted dietary inflammatory index, *IQR* interquartile range, *DHQ* dietary history questionnaire, *BMI* body mass index, *BCS* breast-conserving surgery, *ER* estrogen receptor, *PR* progesterone receptor, *HER2* human epidermal growth factor receptor 2.

^a^*P* value was calculated from Kruskal–Wallis test.

^b^*P* value was calculated from Chi-Square test.

^c^*P* value was calculated from Fisher’s exact.

^d^Other race including Pacific Islander and American Indian.

^e^Single including never married, divorced, separated, and widowed.

^f^Other surgery including biopsy only and other specify.

### Post-diagnosis dietary inflammatory index and survival of breast cancer patients

**Fig. 2:**
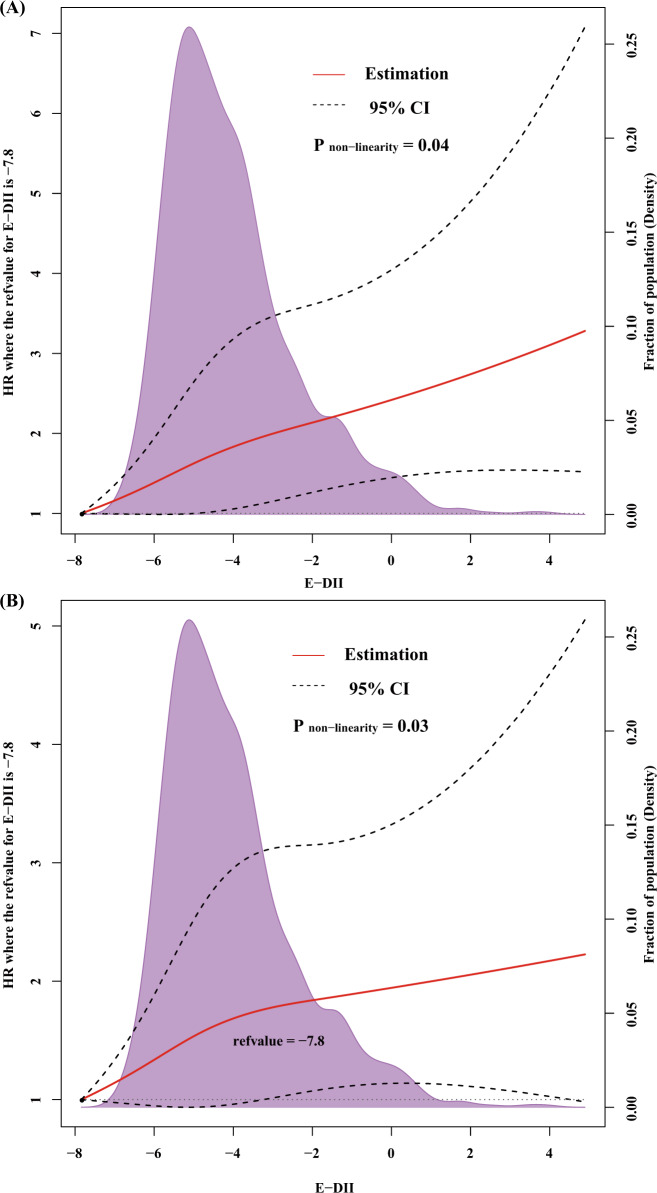
Non-linear dose–response analysis on E-DII scores and all-causes mortality. Non-linear dose-response curves for **a** age and total energy-adjusted and **b** multivariable-adjusted Cox regression model. The reference level was set at −7.9. A *P*_non-linearity_ was obtained by testing the null hypothesis that regression coefficient of the second spline was equal to zero. CI confidence interval.

After the full adjustment for potential confounders, breast cancer patients consuming the most pro-inflammatory diets compared with the most anti-inflammatory diets had a 34% higher risk of death from all-causes based on model2 (HR T3 vs T1, 1.34; 95% CI, 1.01–1.81; *P*_trend_, 0.049, Table 2; seen Supplementary Table 2 on E-DII from food, but without supplement), where protective effect of an anti-inflammatory diet was also observed (HR for 1-unit increment: 1.06; 95% CI: 1.00–1.13, Table 2) when E-DII was treated as a continuous variable. However, there was no statistically significant association between the E-DII and breast cancer-specific mortality in breast cancer patients in the multivariable-adjusted competing risk regression model (HR T3 vs T1, 1.47; 95% CI, 0.89–2.43; *P*_trend_, 0.13, Table 3; seen Supplementary Table 3 on E-DII from food, but without supplement), we still found a protective effect of anti-inflammatory diet when considering E-DII as a continuous variable (multivariable-adjusted HR for 1-unit increment: 1.10; 95% CI: 1.00–1.22, Table 3). When we excluded cases with less than 1 year from breast cancer diagnosis to DHQ completion (*n* = 454), both the HRs for all-cause mortality and breast cancer-specific mortality in model2 became much stronger (for all-causes mortality, HR T3 vs T1 1.66; 95% CI,1.12–2.46, Table 2; for breast cancer-specific mortality, HR T3 vs T1 2.20; 95% CI, 1.14–4.25, Table 3). The results of sensitivity analyses on 1–2 or >2 years before completing the DHQ were reported in Supplementary Table 4. Dose–response analyses found that E-DII scores were positively associated with risk of all-cause mortality in a non-linear dose–response manner (Fig. 2).

**Table 2:** Association between E-DII from food plus supplement and all-causes mortality risk among 1064 breast cancer cases in the PLCO Cancer Screening Trial.

Tertile of E-DII score	Death from any cause (*n*)	Person-years	Hazard ratio (95% confidence interval)	
			Model^a^	Mode2^b^
Tertile 1 (−7.8, −5.6)	86	4942	1.00 (reference)	1.00 (reference)
Tertile 2 (−5.6, −4.1)	98	4888	1.14 [0.85, 1.52]	1.16 [0.87, 1.56]
Tertile 3 (−4.1, 4.9)	112	4477	1.58 [1.19, 2.09]	1.34 [1.01, 1.81]
*P*_trend_			0.002	0.049
Per 1-unit DII increment	296	14,307	1.10 [1.04, 1.17]	1.06 [1.00, 1.13]
After excluding cases with less 1 year from breast cancer diagnosis to DHQ completion^c^				
Tertile 1 (−7.8, −5.6)	17	3400	1.00 (reference)	1.00 (reference)
Tertile 2 (−5.6, −4.1)	15	3280	0.89 [0.45, 1.76]	0.99 [0.73, 2.05]
Tertile 3 (−4.1, 4.9)	25	2691	1.66 [0.90, 3.05]	2.20 [1.14, 4.25]
* P*_trend_			0.08	0.03
Per 1-unit DII increment	172	9371	1.21[1.08, 1.35]	1.21 [1.06, 1.39]

^a^Adjusted for age of breast cancer diagnosis (continues) and total energy intake (continues, kcal/day).

^b^Stratified by age of breast cancer diagnosis (≤60 years old, >60 years old), years from breast cancer diagnosis to DHQ completion (≤1 year, >1 year), stage (0/I, II/III) due to PH assumption violation and adjusted for total energy intake (continues, kcal/day), body mass index (continues, kg/m^2^), trial arm (control, intervention), race (white, black, others), marital status (single, married, divorced or separated, widowed), income(<$20,000, $20,000–$49,000, $50,000–$99,000, $100,000–$200,000, <$200,000), educational level (less than high school, high school graduate or equivalent, post-high school education, college education or higher), smoking status (never smoked, past smoked, current smoked), hormone replacement therapy (never used, former used, current used), history of diabetes (no, yes), physical activity (active less than one time per month, active at least one time per month), estrogen receptor status (negative, positive), and progesterone receptor status (negative, positive).

^c^Model included 610 cases and was adjusted for covariates listed in b except years from breast cancer diagnosis to DHQ completion.

**Table 3:** Association between E-DII from food plus supplement and breast cancer-specific mortality risk among 1064 breast cancer cases in the PLCO Cancer Screening Trial.

Tertile of E-DII score	Death from breast cancer (*n*)	Person-years	Sub-distribution hazard ratio (95% confidence interval)	
			Model^a^	Model^b^
Tertile 1 (−7.8, −5.6)	28	4942	1.00 (reference)	1.00 (reference)
Tertile 2 (−5.6, −4.1)	26	4888	0.91 [0.53, 1.54]	0.88 [0.51, 1.53]
Tertile 3 (−4.1, 4.9)	46	4477	1.73 [1.09, 2.74]	1.47 [0.89, 2.43]
*P*_trend_			0.02	0.13
Per 1-unit DII increment	100	14,307	1.16 [1.06, 1.27]	1.10 [1.00, 1.22]
After excluding cases with less 1 year from breast cancer diagnosis to DHQ completion^c^				
Tertile 1 (−7.8, −5.6)	17	3400	1.00 (reference)	1.00 (reference)
Tertile 2 (−5.6, −4.1)	15	3280	0.89 [0.45, 1.76]	0.99 [4.73, 2.05]
Tertile 3 (−4.1, 4.9)	25	2691	1.66 [0.90, 3.05]	2.20 [1.14, 4.25]
* P*_trend_			0.08	0.03
Per 1-unit DII increment	172	9371	1.21[1.08, 1.35]	1.21 [1.06, 1.39]

^a^Adjusted for age of breast cancer diagnosis (continues) and total energy intake (continues, kcal/day).

^b^Adjusted for age of breast cancer diagnosis (continues), years from breast cancer diagnosis to DHQ completion (continues) total energy intake (continues, kcal/day), body mass index (continues, kg/m^2^), trial arm (control, intervention), race (white, black, others), marital status (single, married, divorced or separated, widowed), income(<$20,000, $20,000–$49,000, $50,000–$99,000, $100,000–$200,000, <$200,000), educational level (less than high school, high school graduate or equivalent, post-high school education, college education or higher), smoking status (never smoked, past smoked, current smoked), hormone replacement therapy (never used, former used, current used), history of diabetes (no, yes), physical activity (active less than one time per month, active at least one time per month), stage (0/I, II/III), estrogen receptor status (negative, positive), and progesterone receptor status (negative, positive).

^c^Mode2 included 610 cases and was adjusted for covariates listed in b except years from breast cancer diagnosis to DHQ completion.

### Stratified analysis

After stratifying by clinically relevant co-variables, the association between E-DII scores and deaths from all-causes differed only in the subgroups of follow-up time (*P*_interaction_ < 0.001). Women in the most pro-inflammatory diet (T3) subgroup with more than 15 person-years follow-up had a 2.18-fold higher risk of all-cause death than those with the most anti-inflammatory diet (HR T3 vs T1 2.18; 95% CI, 1.03–4.64; *P*_trend_, 0.03, Table 4); whereas no statistically association between E-DII and all-cause mortality was seen among cases with less than 15 person-years follow-up (HR T3 vs T1 1.25; 95% CI, 0.91–1.72; *P*_trend_, 0.18, Table 4).

**Table 4:** Risk of all-causes mortality stratified by follow-up time of breast cancer survivors across tertiles of post-diagnosis E-DII from food plus supplement in the PLCO Cancer Screening Trial.

	E-DII tertile 1 (−7.8, −5.6)	E-DII tertile 2 (−5.6, −4.1)	E-DII tertile 3 (−4.1, 4.9)	*P*_trend_	*P*_interaction_^a^
≤15 person-years, (*n*)	180	172	216		<0.001
Death from any cause (*n*)	70	79	93		
Age and energy-adjusted HR (95% CI)	1.00 (reference)	1.19 [0.87, 1.65]	1.27 [0.93, 1.73]	0.14	
Multivariable-adjusted HR (95% CI)^b^	1.00 (reference)	1.21 [0.87, 1.69]	1.25 [0.91, 1.72]	0.18	
>15 person-years (*n*)	175	182	139		
Death from any cause (*n*)	16	19	19		
Age and energy-adjusted HR (95% CI)	1.00 (reference)	1.29 [0.66, 2.52]	2.06 [1.05, 4.05]	0.04	
Multivariable-adjusted HR (95% CI)^b^	1.00 (reference)	1.24 [0.61, 2.54]	2.18 [1.03, 4.64]	0.04	

^a^*P*_interaction_ was calculated by adding the cross-product of quartile E-DII and the follow-up time (≤15 person years and >15 person-years) in the COX proportional hazards regression model.

^b^Stratified by age of breast cancer diagnosis (≤60 years old, >60 years old), years from breast cancer diagnosis to DHQ completion (≤1 year, >1 year), stage (0/I, II/III) due to PH assumption violation and adjusted for total energy intake (continues, kcal/day), body mass index (continues, kg/m2), trial arm (control, intervention), race (white, black, others), marital status (single, married, divorced or separated, widowed), income(<$20,000, $20,000–$49,000, $50,000–$99,000, $100,000–$200,000, <$200,000), educational level (less than high school, high school graduate or equivalent, post-high school education, college education or higher), smoking status (never smoked, past smoked, current smoked), hormone replacement therapy (never used, former used, current used), history of diabetes (no, yes), physical activity (active less than one time per month, active at least one time per month), estrogen receptor status (negative, positive), and progesterone receptor status (negative, positive).

## Discussion

Overall, in this longitudinal analysis of post-menopausal breast cancer patients from the PLCO trial, a more anti-inflammatory diet after breast cancer diagnosis was associated with lower risks of both all-cause mortality and breast cancer-specific mortality. These risks varied by follow-up period, protective effects of consuming anti-inflammatory diet on prognosis of breast cancer became stronger when cases after long-term follow-up.

At present, only one prospective study has evaluated the influence of post-diagnosed dietary inflammatory potential on breast cancer outcomes^18^. In contrast to our findings, Zheng et al. found that among invasive breast cancer patients the E-DII was associated with cardiovascular disease mortality rather than all-cause or breast cancer-specific survival. That study had a slightly larger sample size but a shorter follow-up period than ours. Nevertheless, our study had a broader range of variability in the E-DII (−7.8 to 4.9) than theirs (−6.8 to 3.8). Another retrospective study found anti-inflammatory diets might improve the survival of breast cancer patients, particularly among younger women, those who were premenopausal, obese, had HR + breast cancer, had tumor more than 2 cm, and had lymph node metastasis^7^. However, they used the DII (not the E-DII) and did not consider dietary supplements^19^. Three studies^29–31^ considered other cancer types in relation to post-diagnosis E-DII score on the risk of mortality, however, a post-diagnosis proinflammatory diet was not statistically significantly associated with mortality risk in cancer patients. Relatively short-term follow-up time and retrospective study designs maybe contribute to above-mentioned null results, which are similar to what we found when we examined the short duration (≤15 person-years) subgroup. Further studies with extended follow-up time will be warranted in the future.

Prior studies have suggested that patients tend to make changes in their diet after a cancer diagnosis^32,33^. A previous study reported that dietary modifications were observed in women with breast cancer in Malaysia, and 66.7% were found to have decreased intake of energy, protein, total fat and vitamin E, and increased intake of carotene and vitamin C intake^34^. A prospective analysis from the Women’s Health Initiative^35^ reported that dietary inflammatory potential before diagnosis is related to breast cancer death; however, future studies are needed to examine the inflammatory potential of post-diagnosis diet that is an important approach to conduct dietary intervention in process of the secondary breast cancer prevention. Improved recurrence-free survival and disease-free survival were observed in a large dietary intervention trial among women diagnosed with breast cancer who received dietary fat reduction treatment for breast cancer in the USA^12^. Furthermore, the Nurse’s Health Study (NHS)^36^ with a median of 9.3 years follow-up, including breast cancer patients more than 60 years old, investigated Dietary Approaches to Stop Hypertension (DASH) score and the Alternative Healthy Eating Index (AHEI)-2010 and risks of deaths from breast cancer and all causes. This NHS study reported better adherence to a priori dietary indices after breast cancer diagnosis was associated with a 28% (DASH) and 43% (AHEI) reduced risk of non-breast cancer mortality. The WHI reported that in breast cancer survivors, postdiagnosis higher Healthy Eating Index (HEI)-2005 scores that reflected better quality diets were associated with better overall and cause-specific survival^37^. Because a lower E-DII score, indicating a more anti-inflammatory diet pattern, is associated with better diet quality score (i.e., DASH, AHEI, and HEI), our results are consistent with what was observed in those studies.

We did not find any interaction effects of ER/PR status on the association between E-DII score and risk of all-cause mortality, perhaps due to limited sample size and resulting loss in statistical power. A prior WHI study indicated that a positive relationship between E-DII scores and all-cause mortality risk was seen among ER-positive breast cancer cases and among the combined ER-positive and/or PR-positive cases but not ER-negative /PR-negative cases^38^. In another WHI study, better dietary quality also was associated with a reduced risk of all-cause mortality among women with ER-positive tumors rather than ER-negative tumors^37^. Due to unbalanced distribution of the incidence rate and significantly different prognosis of ER-positive and ER-negative tumors^39^, we suggest that further experimental and epidemiological studies are warranted to validate associations between diet and breast cancer subtypes.

We failed to show that the effect of the E-DII on all-cause mortality in breast cancer survivors differed between smokers and non-smokers; a study focusing on high-grade serous ovarian carcinoma found a protective effect of anti-inflammatory diet on all-cause mortality risk among smokers^31^. Biologically, cigarette smoke contains many oxidants and free radicals and pro-inflammatory compounds that may activate endogenous mechanisms such as recruitment of neutrophils and macrophages to further increase the oxidant injury^40^. The shift in balance between oxidant/antioxidant in favor of oxidants, termed “oxidative stress”, results in many pathological conditions including cancers^41^. Thus, anti-inflammatory diets, including supplements such as vitamins C and E), and β-carotene could protect smokers from experiencing oxidative stress^41^.

The strengths of this study include its prospective cohort design, the standardized dietary assessment using a FFQ that covered major foods and nutrients consumed by Americans, detailed co-variables, long follow-up period, and the use of E-DII that is a comprehensive assessment of dietary inflammatory from food plus supplement. Of note, this is the first study to indicate that a more anti-inflammatory diet after breast cancer diagnosis is associated with both better overall survival and breast cancer-specific survival. Sensitivity analyses and stratified analyses were conducted to highlight the stability of our results. Despite its strengths, several limitations should be noted. First, nearly 50% (2319 of 4561) of otherwise eligible women were excluded because they had a cancer diagnosis before completing the DHQ. In this process of exclusions, some selection bias might exist. Second, related to this first issue, the number of deaths was relatively small, thus precluding stratified analyses with sufficient statistical power to observe significant associations, especially in subgroups for breast cancer-specific mortality. We failed to detect a meaningful association between E-DII and the few observed cardiovascular disease mortality events, even though there is considerable evidence showing that consuming a more anti-inflammatory diet after breast cancer diagnosis can reduce the risk of death from cardiovascular disease^18^. Third, the FFQ in the PLCO provided only 35 out of 45 DII components, which might lead to under- or over-estimation of the relationship of E-DII with mortality^17^. In reality, we had demonstrated that missing even 15–20 parameters had little effect on DII scores in our prior work^42^. Fourth, the FFQ is known to suffer from a variety of biases associated with structured questionnaires. Most prominent among these are response sets such as social approval and social desirability^43,44^. This leads to the fifth weakness, which is that we had no information on any of these potentials in biasers^45,46^. Missing information on HER2 status and adherence to treatments resulted in missing data in the multivariable models, with the potential to bias HR estimates. Participants were excluded who did not have valid DHQ responses. This could potentially bias the results. When analyzing subjects who were excluded from the current analysis due to an invalid or missing DHQ, we found that they were likely to be older, black, obese, divorced or widowed, have physical activity less than one time per month, and experience hormone therapy than those who were included in the risk estimates (Supplementary Table 5), indicating that the results of our study should be extrapolated to the US breast cancer survivors with caution.

In conclusion, this study evaluated the association of the inflammatory potential of diet with all-causes and breast cancer-specific mortality risks in a prospective cohort study of breast cancer survivors. Our findings support that anti-inflammatory potential of a post-diagnosis diet may be a means for reducing risk of breast cancer and all-causes death among breast cancer survivors. To precisely tailor the dietary interventions for breast cancer survivors in the future, additional well-designed cohort studies with large number of cases are warranted to validate our results and identify specific subgroups who would have a survival benefit of post-diagnosis anti-inflammatory diets.

## Methods

### Study design

PLCO was sponsored by the National Cancer Institute (NCI), conducted in the United States, and its purpose was to determine the effects of screening on cancer-related mortality and secondary endpoints^47^. In total, 154,897 eligible participants (76,682 males and 78,215 females), aged 55–74 years were enrolled from November 1993 to July 2001^47^. Participants were individually randomized to the intervention group or the control group in equal proportions. Data were collected on cancer diagnoses and deaths from all causes occurred through July 31, 2011.

### Ethics section

All the participants provided written informed consent to participate in the study, and the study protocol (https://biometry.nci.nih.gov/cdas/plco/) was approved by the Institutional Review Board of the United States National Cancer Institute (NCI).

### Study population

To extract non-metastatic breast cancer patients who had valid DHQ responses after breast cancer diagnosis, our analytical cohort initially identified 4561 post-menopausal women who developed a first-primary breast cancer (International Classification of Diseases for Oncology, Third Edition, codes C50.0-C50.6, C508-C509). According to the exclusion criteria, we removed from consideration 2319 women whose dietary information were collected prior to their breast cancer diagnosis; 1017 cases with invalid DHQ responses (i.e., valid DHQ responses were defined as having DHQ completion date; alive at DHQ completion; <8 missing DHQ responses; and plausible caloric intake defined as within the sex-specific first and last percentiles of total energy); 14 cases who did not return baseline questionnaires and 147 cases with IV stage or unknown stage tumor. After these exclusions, the analytical cohort included 1064 breast cancer patients with follow-up data.

### Dietary assessment

Dietary assessment was similar to our previous study^48^. Diet was assessed by a self-reported food frequency questionnaire (FFQ), the DHQ version 1.0 (National Cancer Institute, 2007) introduced in 1998 to the control and intervention arms within a median of three years after randomization in this trial^49,50^. On the DHQ, participants reported their frequency of intake and portion size of 124 food items and supplement use over the previous year^50,51^. Daily nutrient intake was calculated by the DietCalc software^52^, which integrated responses of food frequency, portion size, and other responses with a nutrient database based on national dietary data (USDA’s 1994–96 Continuing Survey of Food Intakes by Individuals and supplemented by the Nutrition Data Systems for Research from the University of Minnesota)^49^. The DHQ has been validated against four 24-h dietary recalls (one in each season) among 1640 nationally representative participants in the Eating at America’s Table Study where the energy-adjusted correlation coefficients for dietary factors ranged from 0.51 for vitamin E to 0.78 for magnesium among women and from 0.41 for sodium to 0.83 for thiamin among men^51^.

### Energy-adjusted DII (E-DII^TM^) score calculation

**Fig. 3:**
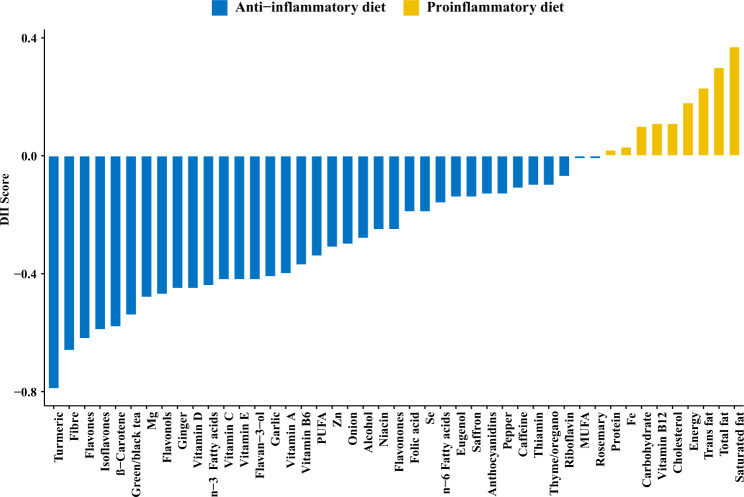
Food parameter-specific DII score. The DII score distribution according anti-inflammatory and proinflammatory diets.

The DII is a literature-derived, population-based index designed to estimate the overall inflammatory potential of an individual’s diet. The details of the development of the DII have been published previously^17^. The energy-adjusted DII (E-DII) score was calculated based on reported nutrient and food intake from the DHQ which were linked to the corresponding inflammatory effect scores designated in the DII^17,53^ (Fig. 3 and Supplementary Fig. 2). Briefly, 1943 eligible peer-reviewed primary research articles incorporating cell culture, animal and epidemiological studies published up to 2010 on the effect of dietary factors on six inflammatory markers (interleukin (IL)-1β, IL-4, IL-6, IL-10, tumor necrosis factor- alpha (TNF-α), and C-reactive protein (CRP)) were identified and scored to derive the component-specific inflammatory effect scores for 45 dietary factors (i.e., components of DII), which comprised macronutrients, micronutrients and some foods or bioactive components such as spices and tea^17^.

Eight DII components including ginger, turmeric, garlic, oregano, rosemary, eugenol, saffron, and flavonols were not available from the DHQ. The remaining 37 components were available for E-DII score calculation in our analysis. The food and nutrient consumption estimated at the DHQ was first adjusted for total energy per 1000 calories. To avoid the arbitrariness as a result of simply using raw intake amounts, the energy-adjusted dietary intake was subsequently standardized to a composite dietary database representing energy-adjusted dietary intake from 11 populations living in different countries across the world^17,54^. The energy-adjusted standardized dietary intake was then multiplied by the literature-derived inflammatory effect score for each DII component, and summed across all components to obtain the overall E-DII score^17^. Higher E-DII scores represent more pro-inflammatory diets while lower (i.e., more negative) E-DII scores indicate more anti-inflammatory diets. The DII score has been construct-validated with different dietary assessment instruments and found to be associated with higher levels of IL-6^55^, high-sensitivity CRP^42^, and homocysteine^56^. Because most of the participants (79%) in the PLCO consumed supplements, and many dietary factors used in supplements have anti-inflammatory properties^57^, we report E-DII from food plus supplements.

### Covariate assessments

Information on age at breast cancer diagnosis, race/ethnicity, marital status, education level, smoking status, income level, number of living birth, physical activity, breast feeding, oophorectomy status, family history of breast cancer and history of diabetes were assessed at baseline using self-administered questionnaires. Hormone therapy, birth control pills, aspirin use and total energy intake were acquired from DHQ. BMI was calculated as weight (kg)/height(m)^2^ and categorized based on the World Health Organization criteria. Years from breast cancer diagnosis to DHQ completion was defined as interval time between breast cancer diagnosis and DHQ completion.

Detailed cancer characteristics such as diagnosis date, stage, tumor morphology (behavior, grade) and hormone receptor results were acquired. The cutoff for estrogen receptor (ER)-negative and progesterone receptor (PR)-negative IHC status was less than 1% staining in the nuclei.

### Ascertainment of death

Outcomes included death from breast cancer and any cause. As previously described^58^, death was primarily ascertained through a mailed annual study update questionnaire, with next of kin notifying the trial of death, which were verified by obtaining death certificates; searches of the National Death Index also were conducted to ascertain death. Autopsy and hospitalization records were used to determine the underlying cause of death. If these were unavailable, death certificates, medical records or other records were utilized.

### Ethical approval

All procedures performed in studies involving human participants were in accordance with the ethical standards of the institutional and/or national research committee and with the 1964 Helsinki declaration and its later amendments or comparable ethical standards.

### Informed consent

Informed consent was obtained from all individual participants included in the PLCO study.

### Statistical analysis

We divided the eligible patients into tertiles according to E-DII from food plus supplements with cutoff points determined from the distribution of the entire cohort. To present the baseline characteristics of the study cases, median ((interquartile range (IQR)) for continuous variables that are not normally distributed as indicated by Shapiro–Wilk normality test (all *P* < 0.05) and frequencies (percentages) for categorical variables was calculated. Accordingly, Kruskal–Wallis test and Chi-Square test (Fisher’s exact if needed) were employed to test differences of continuous and categorical-co-variates, respectively, between the three groups.

For each mortality outcome, women were followed from diagnosis of primary invasive breast cancer until death, loss to follow-up or the end of follow-up. Cox proportional modeling was fitted to estimate crude, age and total energy intake-adjusted and multivariable-adjusted HRs and 95% confidence intervals (CI) for breast cancer patients in the lowest E-DII tertile (most anti-inflammatory diet) as the referent. To minimize the potential impacts of competing risk bias on the association of E-DII with breast cancer-specific mortality risk, competing risk regression models were employed to estimate sub-distribution HR and 95% CIs, with non-breast cancer causes of death as competing risk events^59^. We tested a linear trend across tertile of E-DII using median E-DII value of each tertile, which was regarded as a continuous variable in regression analyses. Additionally, continuous E-DII variable was used to estimate risk estimates per 1-unit increment. Variables were considered as confounding factors if they were associated with both mortality risk and E-DII (in either continuous or categorical format) or they changed the crude risk estimate by >10% in bivariate analyses^60^, in addition, prognostic factors for breast cancer patients from literature review also were treated as confounding factors. In model 1, we adjusted for age at time of breast cancer diagnosis and total energy intake. Model 2 additionally adjusted for years from breast cancer diagnosis to DHQ completion, body mass index, trial arm, race, marital status, income, educational level, hormone replacement therapy, history of diabetes, physical activity, stage, and ER/PR status. Cancer stage and ER/PR status were used as substitutes for the currently unavailable cancer treatment data, as breast cancer stage and hormone receptor status may influence types of treatment received^61,62^. The proportional hazards (PH) assumption was examined using the Schoenfeld residual test^63^, and we only find that co-variables like age, years from breast cancer diagnosis to DHQ completion and stage violated the PH assumption in multivariable analyses. Thus, we fitted an extended Cox proportional hazards models stratified by age (≤60 years old, >60 years old), years from breast cancer diagnosis to DHQ completion (≤1 year, >1 year) and stage (0/I, II/III).

Effect modification by baseline characteristics and clinicopathological variables was examined by adding the cross-product of each effect modifier with E-DII tertile in the multivariable-adjusted model. Likelihood ratio tests were conducted, and *p* values < 0.05 were considered as an indicator of significant effect modification. We planned a priori stratified analysis by important co-variables that were considered clinically relevant, including age (≤60, >60 years), tumor stage (carcinoma in situ and invasive), smoking status (never smoker, former smoker, current smoker), ER/PR status and follow-up time (≤15 person-years, >15 person-years) whose median value is 15 person-years on the association between E-DII and all-cause mortality. Given the small number of breast cancer-specific death in this study, we did not further stratify analyses of breast cancer-specific mortality in interaction analyses.

We further assessed the potential non-linear dose–response relationship of E-DII to all-causes mortality through restricted cubic spline models with 3 knots at the 10th, 50th, and 90th percentiles^64^, and the reference level was set at −7.9 (the lowest value of E-DII in this study). Specifically, a *P*_non-linearity_ was obtained by testing the null hypothesis that the regression coefficient of the second spline was equal to zero.

In sensitivity analyses, considering dietary changes due to adjuvant therapies involving chemotherapy and radiotherapy for the disease might affect appetite^65^, we excluded cases with less than 1 year from breast cancer diagnosis to DHQ completion.

All P values reported are two-sided. Those less than 0.05 were considered statistically significant. All analyses were conducted using R software (version 3.4.1).

### Reporting summary

Further information on research design is available in the Nature Research Reporting Summary linked to this article.

## Supplementary information

Supplementary Information

Reporting Summary

## Data Availability

Clinical and dietary data that support the findings of this study have been deposited in PLCO trial (https://biometry.nci.nih.gov/cdas/plco/), and PLCO has the following five ClinicalTrials.gov registration numbers: NCT00002540 (Prostate), NCT01696968 (Lung), NCT01696981 (Colorectal), NCT0169699 (Ovarian), and NCT00339495 (EEMS). A metadata record describing the underlying data, its availability and usage terms is available in the figshare repository: 10.6084/m9.figshare.12605924.^66^

## Code availability

The code for data processing and visualization is written in R and available upon request (K.W: kang_wang0822@hotmail.com).

## References

[1] Miller KD (2019). Cancer treatment and survivorship statistics, 2019. CA Cancer J. Clin..

[2] Goss PE (2014). Challenges to effective cancer control in China, India, and Russia. Lancet Oncol..

[3] Berry DA (2005). Effect of screening and adjuvant therapy on mortality from breast cancer. N. Engl. J. Med..

[4] Ma J (2019). The American Cancer Society 2035 challenge goal on cancer mortality reduction. CA Cancer J. Clin..

[5] Klassen AC (2018). “We’re Just Not Prepared for Eating Over Our Whole Life”: a mixed methods approach to understanding dietary behaviors among longer term cancer survivors. Integr. Cancer Ther..

[6] Springfield S, Odoms-Young A, Tussing-Humphreys L, Freels S, Stolley M (2019). Adherence to American Cancer Society and American Institute of Cancer Research dietary guidelines in overweight African American breast cancer survivors. J. Cancer Survivorship Res. Pract..

[7] Burden, S., Sremanakova, J., Jones, D. & Todd, C. Dietary interventions for cancer survivors. *Proc. Nutr. Soc.* 1–11, 10.1017/S0029665118002690 (2018). 10.1017/S0029665118002690 30563580

[8] Dieli-Conwright CM (2018). Adipose tissue inflammation in breast cancer survivors: effects of a 16-week combined aerobic and resistance exercise training intervention. Breast Cancer Res. Treat..

[9] Nagahashi M (2018). Targeting the SphK1/S1P/S1PR1 axis that links obesity, chronic inflammation, and breast cancer metastasis. Cancer Res..

[10] DeNardo DG, Coussens LM (2007). Inflammation and breast cancer. Balancing immune response: crosstalk between adaptive and innate immune cells during breast cancer progression. Breast Cancer Res. BCR.

[11] Pierce BL (2009). Elevated biomarkers of inflammation are associated with reduced survival among breast cancer patients. J. Clin. Oncol. Off. J. Am. Soc. Clin. Oncol..

[12] Chlebowski RT (2006). Dietary fat reduction and breast cancer outcome: interim efficacy results from the Women’s Intervention Nutrition Study. J. Natl. Cancer Inst..

[13] Pierce JP (2007). Influence of a diet very high in vegetables, fruit, and fiber and low in fat on prognosis following treatment for breast cancer: the Women’s Healthy Eating and Living (WHEL) randomized trial. JAMA.

[14] McCullough ML (2016). Pre- and postdiagnostic diet in relation to mortality among breast cancer survivors in the CPS-II Nutrition Cohort. Cancer Causes Control. CCC.

[15] Chlebowski RT (2017). Low-fat dietary pattern and breast cancer mortality in the women’s health initiative randomized controlled trial. J. Clin. Oncol. Off. J. Am. Soc. Clin. Oncol..

[16] Krebs-Smith SM, Subar AF, Reedy J (2015). Examining dietary patterns in relation to chronic disease: matching measures and methods to questions of interest. Circulation.

[17] Shivappa N, Steck SE, Hurley TG, Hussey JR, Hébert JR (2014). Designing and developing a literature-derived, population-based dietary inflammatory index. Public Health Nutr..

[18] Zheng J (2018). Association between post-cancer diagnosis dietary inflammatory potential and mortality among invasive breast cancer survivors in the women’s health initiative. Cancer Epidemiol. Biomark. Prev. Publ. Am. Assoc. Cancer Res. Cosponsored Am. Soc. Preventive Oncol..

[19] Jang, H., Chung, M. S., Kang, S. S. & Park, Y. Association between the dietary inflammatory index and risk for cancer recurrence and mortality among patients with breast cancer. *Nutrients* **10**, 10.3390/nu10081095 (2018). 10.3390/nu10081095 PMC6115987 30111758

[20] Zucchetto A (2017). Dietary inflammatory index before diagnosis and survival in an Italian cohort of women with breast cancer. Br. J. Nutr..

[21] Anderson C (2017). Age- and treatment-related associations with health behavior change among breast cancer survivors. Breast.

[22] Li D (2018). Dose-response relation between dietary inflammatory index and human cancer risk: evidence from 44 epidemiologic studies involving 1,082,092 participants. Am. J. Clin. Nutr..

[23] Ge I (2015). Dietary inflammation potential and postmenopausal breast cancer risk in a German case-control study. Breast.

[24] Coughlin SS, Paxton RJ, Moore N, Stewart JL, Anglin J (2019). Survivorship issues in older breast cancer survivors. Breast Cancer Res. Treat..

[25] Fung TT (2006). Diet quality is associated with the risk of estrogen receptor-negative breast cancer in postmenopausal women. J. Nutr..

[26] Mtintsilana, A. et al. Adiposity mediates the association between the dietary inflammatory index and markers of type 2 diabetes risk in middle-aged black South African women. *Nutrients* **11**, 10.3390/nu11061246 (2019). 10.3390/nu11061246 PMC6628082 31159253

[27] Yap, R. W. K., Shidoji, Y., Yap, W. S. & Masaki, M. Association and interaction effect of AGTR1 and AGTR2 gene polymorphisms with dietary pattern on metabolic risk factors of cardiovascular disease in Malaysian adults. *Nutrients* **9**, 10.3390/nu9080853 (2017). 10.3390/nu9080853 PMC5579646 28792482

[28] Alkerwi A (2017). Smoking status is inversely associated with overall diet quality: findings from the ORISCAV-LUX study. Clin. Nutr..

[29] Ratjen I (2019). Association between the dietary inflammatory index and all-cause mortality in colorectal cancer long-term survivors. Int. J. Cancer.

[30] Zucchetto A (2016). Dietary inflammatory index and prostate cancer survival. Int. J. Cancer.

[31] Peres LC (2019). Prediagnostic proinflammatory dietary potential is associated with all-cause mortality among African-American women with high-grade serous ovarian carcinoma. J. Nutr..

[32] Alfano CM (2009). Exercise and dietary change after diagnosis and cancer-related symptoms in long-term survivors of breast cancer: CALGB 79804. Psycho-Oncol..

[33] Rabin C, Pinto B (2006). Cancer-related beliefs and health behavior change among breast cancer survivors and their first-degree relatives. Psycho-Oncol..

[34] Shaharudin SH, Sulaiman S, Shahril MR, Emran NA, Akmal SN (2013). Dietary changes among breast cancer patients in Malaysia. Cancer Nurs..

[35] Tabung FK (2016). Association between dietary inflammatory potential and breast cancer incidence and death: results from the Women’s Health Initiative. Br. J. Cancer.

[36] Izano MA, Fung TT, Chiuve SS, Hu FB, Holmes MD (2013). Are diet quality scores after breast cancer diagnosis associated with improved breast cancer survival?. Nutr. Cancer.

[37] George SM (2014). Better postdiagnosis diet quality is associated with reduced risk of death among postmenopausal women with invasive breast cancer in the women’s health initiative. Cancer Epidemiol. Biomark. Prev..

[38] J Z (2018). Association between post-cancer diagnosis dietary inflammatory potential and mortality among invasive breast cancer survivors in the women’s health initiative. Cancer Epidemiol. Biomark. Prev. Publ. Am. Assoc. Cancer Res. Cosponsored Am. Soc. Prevent. Oncol..

[39] Bray F (2018). Global cancer statistics 2018: GLOBOCAN estimates of incidence and mortality worldwide for 36 cancers in 185 countries. CA Cancer J. Clin..

[40] Church DF, Pryor WA (1985). Free-radical chemistry of cigarette smoke and its toxicological implications. Environ. Health Perspect..

[41] Wang M (1996). Lipid peroxidation-induced putative malondialdehyde-DNA adducts in human breast tissues. Cancer Epidemiol. Biomark. Prev. Publ. Am. Assoc. Cancer Res. Cosponsored Am. Soc. Prevent. Oncol..

[42] Shivappa N (2014). A population-based dietary inflammatory index predicts levels of C-reactive protein in the Seasonal Variation of Blood Cholesterol Study (SEASONS). Public Health Nutr..

[43] Hebert JR, Clemow L, Pbert L, Ockene IS, Ockene JK (1995). Social desirability bias in dietary self-report may compromise the validity of dietary intake measures. Int. J. Epidemiol..

[44] Hebert JR (1997). Gender differences in social desirability and social approval bias in dietary self-report. Am. J. Epidemiol..

[45] Hebert JR (2002). Systematic errors in middle-aged women’s estimates of energy intake: comparing three self-report measures to total energy expenditure from doubly labeled water. Ann. Epidemiol..

[46] Hebert JR (2014). Considering the value of dietary assessment data in informing nutrition-related health policy. Adv. Nutr..

[47] Prorok PC (2000). Design of the prostate, lung, colorectal and ovarian (PLCO) cancer screening trial. Controlled Clin. Trials.

[48] Zheng J (2018). Inflammatory potential of diet and risk of pancreatic cancer in the prostate, lung, colorectal and ovarian (PLCO) cancer screening trial. Int J. Cancer.

[49] Subar AF (2000). Evaluation of alternative approaches to assign nutrient values to food groups in food frequency questionnaires. Am. J. Epidemiol..

[50] Diet History Questionnaire, Version 1.0. *National Institutes of Health, Applied Research Program*, *National Cancer Institute*. https://epi.grants.cancer.gov/DHQ/about/ (2007).

[51] Subar AF (2001). Comparative validation of the Block, Willett, and National Cancer Institute food frequency questionnaires: the Eating at America’s Table Study. Am. J. Epidemiol..

[52] NIH. Diet*Calc Analysis Program, Version 1.4.3. *National Cancer Institute, Applied Research Program*. https://epi.grants.cancer.gov/DHQ/database/ (2005).

[53] N S, SE S, TG H, JR H, JR H (2014). Designing and developing a literature-derived, population-based dietary inflammatory index. Public Health Nutr..

[54] Wirth MD, Shivappa N, Hurley TG, Hebert JR (2016). Association between previously diagnosed circulatory conditions and a dietary inflammatory index. Nutr. Res..

[55] Tabung FK (2015). Construct validation of the dietary inflammatory index among postmenopausal women. Ann. Epidemiol..

[56] Shivappa N (2015). Associations between dietary inflammatory index and inflammatory markers in the Asklepios Study. Br. J. Nutr..

[57] Hamalainen M, Nieminen R, Vuorela P, Heinonen M, Moilanen E (2007). Anti-inflammatory effects of flavonoids: genistein, kaempferol, quercetin, and daidzein inhibit STAT-1 and NF-kappaB activations, whereas flavone, isorhamnetin, naringenin, and pelargonidin inhibit only NF-kappaB activation along with their inhibitory effect on iNOS expression and NO production in activated macrophages. Mediators Inflamm..

[58] Pierre-Victor D, Pinsky PF (2019). Association of nonadherence to cancer screening examinations with mortality from unrelated causes: a secondary analysis of the PLCO cancer screening trial. JAMA Intern. Med..

[59] Lau B, Cole SR, Gange SJ (2009). Competing risk regression models for epidemiologic data. Am. J. Epidemiol..

[60] Maldonado G, Greenland S (1993). Simulation study of confounder-selection strategies. Am. J. Epidemiol..

[61] Fisher B (2004). Treatment of lymph-node-negative, oestrogen-receptor-positive breast cancer: long-term findings from National Surgical Adjuvant Breast and Bowel Project randomised clinical trials. Lancet.

[62] Goldhirsch A (2003). Meeting highlights: updated international expert consensus on the primary therapy of early breast cancer. J. Clin. Oncol..

[63] Schoenfeld D (1980). Chi-squared goodness-of-fit tests for the proportional hazards regression model. Biometrika.

[64] Desquilbet L, Mariotti F (2010). Dose-response analyses using restricted cubic spline functions in public health research. Stat. Med..

[65] Fassier P (2017). Modifications in dietary and alcohol intakes between before and after cancer diagnosis: results from the prospective population-based NutriNet-Sante cohort. Int. J. Cancer.

[66] Wang, K. et al. Metadata supporting the published article: Long-term anti-inflammatory diet in relation to improved breast cancer prognosis: results from a prospective cohort study. *Figshare*. 10.6084/m9.figshare.12605924 (2020). 10.1038/s41523-020-00179-4 PMC7426822 32821804

