# Supplementary material for: Long-term anti-inflammatory diet in relation to improved breast cancer prognosis: a prospective cohort study
Source: NPJ Breast Cancer. 2020 Aug 13;6:36. doi: 10.1038/s41523-020-00179-4 (PMC7426822; doi:10.1038/s41523-020-00179-4)
Supplement: Supplementary file 1 — Supplementary Information [file 41523_2020_179_MOESM1_ESM.pdf]

ONLINE DATA SUPLEMENT TO

Long-term anti-inflammatory diet in relation to improved breast cancer prognosis: a prospective cohort study

BY Kang Wang ET AL

NPJ Breast Cancer

Contents

Supplementary Table 1……………………………………………………………………..2
Supplementary Table 2……………………………………………………………………..7
Supplementary Table 3……………………………………………………………………..8
Supplementary Table 4……………………………………………………………………..9
Supplementary Table 5……………………………………………………………………..10
Supplementary Figure 1…………………………………………………………………......13
Supplementary Figure 2……………………………………………………………………14

Supplementary Table 1 Baseline characteristics of 1,064 breast cancer cases in the PLCO Cancer Screening Trial by tertile of E-DII from food, but without supplement.

	Post-diagnostic exposure to dietary inflammatory potential			
	**E-DII Tertile 1 (-7.3, -4.0)**	**E-DII Tertile 2 (-4.0, -2.1)**	**E-DII Tertile 3 (-2.1, 5.6)**	**P**
**Number of cases**	**355**	**354**	**355**	
	**Median (IQR)**	**Median (IQR)**	**Median (IQR)**	
**Age at breast cancer diagnosis (years)**	65 (61, 70)	66 (62, 70)	65 (60, 70)	0.11 [a]
**Total energy intake (kcal/day)**	1,376 (1,101, 1,753)	1,418 (1,118, 1,766)	1,542 (1,150, 1,923)	0.01 [a]
**Alcohol intake (g/day)**	0.81 (0, 5.0)	0.77 (0, 4.8)	1.23 (0, 6.4)	0.13 [a]
**Years from breast cancer diagnosis to DHQ completion (years)**	1.4 (0.3, 2.5)	1.2 (0.22, 2.2)	1.3 (0.4, 2.1)	0.42 [a]
**Person-years of follow-up since breast cancer diagnosis**	15.0(11.1, 17.2)	14.6 (10.2, 17.0)	14.1 (10.3, 16.4)	0.05 [a]
	**N (%)[b]**	**N (%)[b]**	**N (%)[b]**	
**Trial arm**				
Intervention	189 (53.2)	182 (51.4)	196 (55.2)	0.60 [b]
Control	166 (46.8)	172 (48.6)	159 (44.8)	
**Race/Ethnicity**				
White	311 (87.6)	322 (91.0)	337 (94.9)	0.005 [c]
Black	12 (3.4)	9 (2.5)	12 (3.4)	
Hispanic	3 (0.8)	1 (0.3)	3 (0.8)	
Asian	27 (7.6)	16 (4.5)	3 (0.8)	
Other[d]	2 (0.6)	6 (1.7)	0 (0.0)	
**BMI (kg/m^2)**				
≤18.5	2 (0.6)	4 (1.1)	1 (0.3)	0.003[c]

18.6-25	181 (51.0)	151 (42.7)	134 (37.7)	
26-30	110 (31.0)	127 (35.9)	121 (34.1)	
31-50	62 (17.5)	72 (20.3)	99 (27.9)	
**Marital status**				
Single[e]	17 (4.8)	18 (5.1)	9 (2.5)	0.64[b]
Married or living as married	254 (71.5)	246 (69.5)	259 (73.0)	
Divorced or separated	40 (11.3)	41 (11.6)	37 (10.4)	
Widowed	44 (12.4)	49 (13.8)	50 (14.1)	
**Education level**				
Less than high school	14 (3.9)	18 (5.1)	30 (8.5)	<0.001 [b]
High school graduate or equivalent	59 (16.6)	79 (22.3)	100 (28.2)	
Post-high school education	33 (9.3)	40 (11.3)	42 (11.8)	
College education or higher	249 (70.1)	217 (61.3)	183 (51.5)	
**Income level**				
<$20,000	56 (15.8)	50 (14.1)	64 (18.0)	0.27 [c]
$20,000-$49,000	145 (40.8)	156 (44.1)	162 (45.6)	
$50,000-$99,000	121 (34.1)	126 (35.6)	101 (28.5)	
$100,000-$200,000	27 (7.6)	21 (5.9)	24 (6.8)	
<$200,000	6 (1.7)	1 (0.3)	4 (1.1)	
**Smoking status**				
Never smoked	198 (55.8)	197 (55.6)	169 (47.6)	<0.001 [b]
Past smoker	14 (3.9)	22 (6.2)	46 (13.0)	
Current smoker	143 (40.3)	135 (38.1)	140 (39.4)	
**Physical activity**				
Active less than one time per month	43 (12.1)	49 (13.8)	48 (13.8)	0.77 [b]
Active at least one time per month	312 (87.9)	305 (86.2)	307 (86.5)	

**Hormone therapy**				
Never used	120 (33.8)	117 (33.1)	123 (34.6)	0.78 [b]
Former used	218 (61.4)	220 (62.1)	221 (62.3)	
Current used	17 (4.8)	17 (4.8)	11 (3.1)	
**Birth control pills**				
No	158 (44.5)	168 (47.5)	163 (45.9)	0.73
Yes	197 (55.5)	186 (52.5)	192 (54.1)	
**Aspirin use**				
None	103 (29.0)	89 (25.1)	94 (26.5)	0.28 [b]
< Once/week	76 (21.4)	76 (21.5)	94 (26.5)	
Once per week or more	176 (49.6)	189 (53.4)	167 (47.0)	
**Number of living birth**				
0	34 (9.6)	41 (11.6)	39 (11.0)	0.005 [b]
1-2	150 (42.3)	102 (28.8)	117 (33.0)	
⩾3	171 (48.2)	211 (59.6)	199 (56.1)	
**Breast feeding**				
None or never pregnant	133 (37.5)	150 (42.4)	176 (49.6)	0.04 [b]
<6 months	97 (27.3)	97 (27.4)	89 (25.1)	
6-11 months	63 (17.7)	59 (16.7)	45 (12.7)	
>12 months	62 (17.5)	48 (13.6)	45 (12.7)	
**Oophorectomy status**				
Ovaries not removed				0.62 [b]
removed	63 (17.7)	73 (20.6)	67 (18.9)	
**Family history of breast cancer**				
No	281 (79.2)	287 (81.1)	282 (79.4)	0.41 [c]
Yes	73 (20.6)	63 (17.8)	72 (20.3)	

Possible	1 (0.3)	4 (1.1)	1 (0.3)	
**History of diabetes**				
No	328 (92.4)	327 (92.4)	335 (94.4)	0.49 [b]
Yes	27 (7.6)	27 (7.6)	20 (5.6)	
**Stage**				
In situ	75 (21.1)	72 (20.3)	69 (19.4)	0.47 [c]
I	190 (53.5)	173 (48.9)	172 (48.5)	
II	83 (23.4)	96 (27.1)	104 (29.3)	
III	7 (2.0)	13 (3.7)	10 (2.8)	
**Nuclear grade**				
I	103 (29.0)	95 (26.8)	119 (33.5)	0.39 [b]
II	153 (43.1)	155 (43.8)	146 (41.1)	
III	99 (27.9)	104 (29.4)	90 (25.4)	
**ER status**				
Positive	297 (83.7)	300 (84.7)	303 (85.4)	0.82 [b]
Negative	58 (16.3)	54 (15.3)	52 (14.6)	
**PR status**				
Positive	262 (73.8)	262 (74.0)	276 (77.7)	0.39 [b]
Negative	93 (16.2)	92 (26.0)	79 (22.3)	
**Surgery**				
Lumpectomy	158 (44.5)	148 (41.8)	153 (43.1)	0.91[b]
Mastectomy	152 (42.8)	164 (46.3)	156 (43.9)	
Others[f]	45 (12.7)	42 (11.9)	46 (13.0)	

[a] P value was calculated from Kruskal–Wallis test.

[b] P value was calculated from Chi-Square test.

[c] P value was calculated from Fisher's exact.

[d] Other race including Pacific Islander and American Indian.
[e] Single including never married, divorced, separated and widowed.
[f] Other surgery including biopsy only and other specify.
Abbreviations: E-DII, energy-adjusted dietary inflammatory index; IQR, interquartile range; DHQ, dietary history questionnaire; BMI, body mass index, BCS, breast conserving surgery; ER, estrogen receptor, PR, progesterone receptor, HER2, human epidermal growth factor receptor 2.

Supplementary Table 2. Association between E-DII from food, without supplement and all-causes mortality risk among 1,064 breast cancer cases in the PLCO Cancer Screening Trial.

Tertile of E-DII score	Death from any cause (n)	Person-years	Hazard ratio (95% confidence interval)	
			Model [a]	Mode2 [b]
**Tertile 1 (-7.3, -4.0)**	87	4,911	1.00 (reference)	1.00 (reference)
**Tertile 2 (-4.0, -2.1)**	111	4,705	**1.32 [1.00, 1.75]**	1.25 [0.94, 1.67]
**Tertile 3 (-2.1, 5.6)**	98	4,691	1.25 [0.94, 1.67]	1.15 [0.85, 1.56]
**P_{trend}**			**0.01**	**012**
**Per 1-unit DII increment**	296	14,307	**1.07 [1.02, 1.14]**	**1.05 [0.99, 1.11]**

[a] Adjusted for age of breast cancer diagnosis (continues) and total energy intake (continues, kcal/day).

[b] Stratified by age of breast cancer diagnosis (<=60 years old, >60 years old), years from breast cancer diagnosis to DHQ completion (<=1 year, >1 year), stage (0/I, II/III) due to PH assumption violation and adjusted for total energy intake (continues, kcal/day), body mass index (continues, kg/m^2), trial arm (control, intervention), race (white, black, others), marital status (single, married, divorced or separated, widowed), income(<$20,000, $20,000-$49,000, $50,000-$99,000, $100,000-$200,000, <$200,000), educational level (less than high school, high school graduate or equivalent, post-high school education, college education or higher), smoking status (never smoked, past smoked, current smoked), hormone replacement therapy (never used, former used, current used), history of diabetes (no, yes), physical activity (active less than one time per month, active at least one time per month), estrogen receptor status (negative, positive) and progesterone receptor status (negative, positive).

Supplementary Table 3. Association between E-DII from food, without supplement and breast cancer-specific mortality risk among 1,064 breast cancer cases in the PLCO Cancer Screening Trial.

Tertile of E-DII score	Death from breast cancer (n)	Person-years	Sub-distribution hazard ratio (95% confidence interval)	
			Model [a]	Model [b]
**Tertile 1 (-7.8, -5.6)**	29	4,911	1.00 (reference)	1.00 (reference)
**Tertile 2 (-5.6, -4.1)**	36	4,705	1.25 [0.76, 2.05]	1.05 [0.63, 1.75]
**Tertile 3 (-4.1, 4.9)**	35	4,691	1.20 [0.74, 1.95]	0.98 [0.59, 1.65]
**P_{trend}**			0.18	0.75
**Per 1-unit DII increment**	100	14,307	1.07 [0.97, 1.18]	1.02 [0.91, 1.13]

[a] Adjusted for age of breast cancer diagnosis (continues) and total energy intake (continues, kcal/day).

[b] Adjusted for age of breast cancer diagnosis (continues), years from breast cancer diagnosis to DHQ completion (continues), , total energy intake (continues, kcal/day), body mass index (continues, kg/m^2), trial arm (control, intervention), race (white, black, others), marital status (single, married, divorced or separated, widowed), income(<$20,000, $20,000-$49,000, $50,000-$99,000, $100,000-$200,000, <$200,000), educational level (less than high school, high school graduate or equivalent, post-high school education, college education or higher), smoking status (never smoked, past smoked, current smoked), hormone replacement therapy (never used, former used, current used), history of diabetes (no, yes), physical activity (active less than one time per month, active at least one time per month), stage (0/I, II/III), estrogen receptor status (negative, positive), progesterone receptor status (negative, positive).

Supplementary Table 4. Risk of all-causes mortality stratified by interval from diagnosis to DHQ completion of breast cancer survivors across tertiles of post-diagnosis E-DII from food plus supplement in the PLCO Cancer Screening Trial.

	**E-DII Tertile 1 (-7.8, -5.6)**	**E-DII Tertile 2 (-5.6, -4.1)**	**E-DII Tertile 3 (-4.1, 4.9)**	$\mathbf{P_{trend}}$	$\mathbf{P_{interaction}}$[a]
**1-2 years post-diagnosis**	96	90	88		**0.87**
**Death from any cause (n)**	16	24	24		
**Age and energy-adjusted HR (95% CI)**	1.00 (reference)	1.52 [0.80, 2.87]	**1.91 [1.01, 3.62]**	**0.02**	
**Multivariable-adjusted HR (95% CI)**[b]	1.00 (reference)	1.68 [0.86, 3.26]	**2.10 [1.02, 4.30]**	**0.01**	
**>2 years post-diagnosis**	122	119	95		
**Death from any cause (n)**	34	33	37		
**Age and energy-adjusted HR (95% CI)**	1.00 (reference)	1.02 [0.63, 1.65]	**1.75 [1.09, 2.80]**	**0.03**	
**Multivariable-adjusted HR (95% CI)**[b]	1.00 (reference)	1.09 [0.66, 1.80]	**1.74 [1.04, 2.91]**	0.07	

[a]$P_{interaction}$ was calculated by adding the cross-product of quartile E-DII and the follow-up time (1-2 years and >2 years post-diagnosis) in the COX proportional hazards regression model.

[4] Stratified by age of breast cancer diagnosis (<=60 years old, >60 years old), years from breast cancer diagnosis to DHQ completion (<=1 year, >1 year), stage (0/I, II/III) due to PH assumption violation and adjusted for total energy intake (continues, kcal/day), body mass index (continues, kg/m2), trial arm (control, intervention), race (white, black, others), marital status (single, married, divorced or separated, widowed), income(<$20,000, $20,000-$49,000, $50,000-$99,000, $100,000-$200,000, <$200,000), educational level (less than high school, high school graduate or equivalent, post-high school education, college education or higher), smoking status (never smoked, past smoked, current smoked), hormone replacement therapy (never used, former used, current used), history of diabetes (no, yes), physical activity (active less than one time per month, active at least one time per month), estrogen receptor status (negative, positive) and progesterone receptor status (negative, positive).

Supplementary Table5 Baseline characteristics of breast cancer cases in the PLCO Cancer Screening Trial by status of DHQ response.

	Cohort with invalid DHQ response*	Cohort with valid DHQ response	P
**Number of cases**	**934**	**1,064**	
	**Median (IQR)**	**Median (IQR)**	
**E-DII from food plus supplement**	-3.4 (-5.3, -0.92)	-4.9 (-5.9, -3.6)	<0.001[a]
**Age at breast cancer diagnosis (years)**	69 (64, 74)	65 (61, 70)	<0.001[a]
**Total energy intake (kcal/day)**	1219.4 (722.7, 3730.3)	1435.1 (1119.5, 1820.9)	0.08 [a]
**Alcohol intake (g/day)**	0.17 (0, 1.53)	0.97 (0, 5.2)	<0.001[a]
**Years from breast cancer diagnosis to DHQ completion (years)**	1.4 (0.38, 2.7)	1.3 (0.31, 2.3)	0.23 [a]
**Person-years of follow-up since breast cancer diagnosis**	10.0 (6.8, 13.7)	14.6 (10.5, 16.9)	<0.001 [a]
	**N (%)[b]**	**N (%)[b]**	
**Trial arm**			
Intervention	459 (49.1)	567 (53.3)	0.07 [b]
Control	475 (50.9)	497 (46.7)	
**Race/Ethnicity**			
White	774 (82.9)	970 (91.2)	0.005 [c]
Black	79 (8.5)	33 (3.1)	
Hispanic	22 (2.4)	7 (0.7)	
Asian	45 (4.8)	46 (4.3)	
Other[d]	14 (1.5)	8 (0.8)	
**BMI (kg/m^2)**			
≤18.5	11 (1.2)	7 (0.7)	<0.001[c]
18.6-25	317 (33.9)	466 (43.8)	
26-30	336 (36.0)	358 (33.6)	
31-50	270 (28.9)	233 (21.9)	
**Marital status**			
Single[e]	40 (4.3)	44 (4.1)	<0.001 [b]
Married or living as married	578 (61.9)	759 (71.3)	
Divorced or separated	167 (17.9)	118 (11.1)	
Widowed	149 (16.0)	143 (13.4)	
**Education level**			
Less than high school	70 (7.5)	62 (5.8)	0.06 [b]
High school graduate or equivalent	244 (26.1)	238 (22.4)	
Post-high school education	100 (10.7)	115 (10.8)	

College education or higher	520 (55.7)	649 (61.0)	
**Income level**			
<$20,000	214 (22.9)	170 (16.0)	0.001 [c]
$20,000-$49,000	401 (42.9)	463 (43.5)	
$50,000-$99,000	255 (27.3)	348 (32.7)	
$100,000-$200,000	52 (5.6)	72 (6.8)	
<$200,000	12 (1.3)	11 (1.0)	
**Smoking status**			
Never smoked	496 (53.1)	564 (53.0)	0.02 [b]
Past smoker	105 (11.2)	82 (7.7)	
Current smoker	333 (35.7)	418 (39.3)	
**Physical activity**			
Active less than one time per month	165 (17.7)	140 (13.2)	0.006 [b]
Active at least one time per month	769 (82.3)	924 (86.8)	
**Hormone therapy**			
Never used	333 (35.7)	360 (33.8)	0.03 [b]
Former used	540 (57.8)	659 (61.9)	
Current used	61 (6.5)	45 (4.2)	
**Birth control pills**			
No	463 (49.6)	489 (46.0)	0.12 [b]
Yes	471 (50.4)	575 (54.0)	
**Aspirin use**			
None	246 (26.3)	286 (26.9)	0.85 [b]
< Once/week	209 (22.4)	246 (23.1)	
Once per week or more	479 (51.3)	532 (50.0)	
**Number of live babies delivered**			
0	111 (11.9)	114 (10.7)	0.63 [b]
1-2	310 (33.2)	369 (34.7)	
⩾3	513 (54.9)	581 (54.6)	
**Breast feeding**			
None or never pregnant	412 (44.1)	459 (43.1)	0.21 [b]
<6 months	266 (28.5)	283 (26.6)	
6-11 months	116 (12.4)	167 (15.7)	
>12 months	140 (15.0)	155 (14.6)	
**Oophorectomy status**			
Ovaries not removed			0.38 [b]
removed	163 (17.5)	203 (19.1)	
**Family history of breast cancer**			
No	736 (78.8)	850 (79.9)	0.42 [c]
Yes	188 (20.1)	208 (19.5)	

Possible	10 (1.1)	6 (0.6)	
**History of diabetes**			
No			0.99 [b]
Yes	65 (7.0)	74 (7.0)	
**Stage**			
In situ	176 (18.8)	216 (20.3)	0.21 [c]
I	464 (49.7)	535 (50.3)	
II	252 (27.0)	283 (26.6)	
III	42 (4.5)	30 (2.8)	
**Nuclear grade**			
I	280 (30.0)	317 (29.8)	0.99 [b]
II	397 (42.5)	454 (42.7)	
III	257 (27.5)	293 (27.5)	
**ER status**			
Positive	796 (85.2)	900 (84.6)	0.74 [b]
Negative			
**PR status**			
Positive	687 (73.6)	800 (75.2)	0.43 [b]
Negative			
**Surgery**			
Lumpectomy	438 (46.9)	459 (43.1)	0.19 [b]
Mastectomy	396 (42.4)	472 (44.4)	
Others[f]	100 (10.7)	133 (12.5)	

[*] There were 934 of 1,017 breast cancer patients who completed DHQ after breast cancer diagnosis, had invalid DQH response, and had I-III stage tumors.

[a] P value was calculated from Kruskal–Wallis test.

[b] P value was calculated from Chi-Square test.

[c] P value was calculated from Fisher's exact.

[d] Other race including Pacific Islander and American Indian.

[e] Single including never married, divorced, separated and widowed.

[f] Other surgery including biopsy only and other specify.

Abbreviations: E-DII, energy-adjusted dietary inflammatory index; IQR, interquartile range; DHQ, dietary history questionnaire; BMI, body mass index, BCS, breast conserving surgery; ER, estrogen receptor, PR, progesterone receptor, HER2, human epidermal growth factor receptor 2.

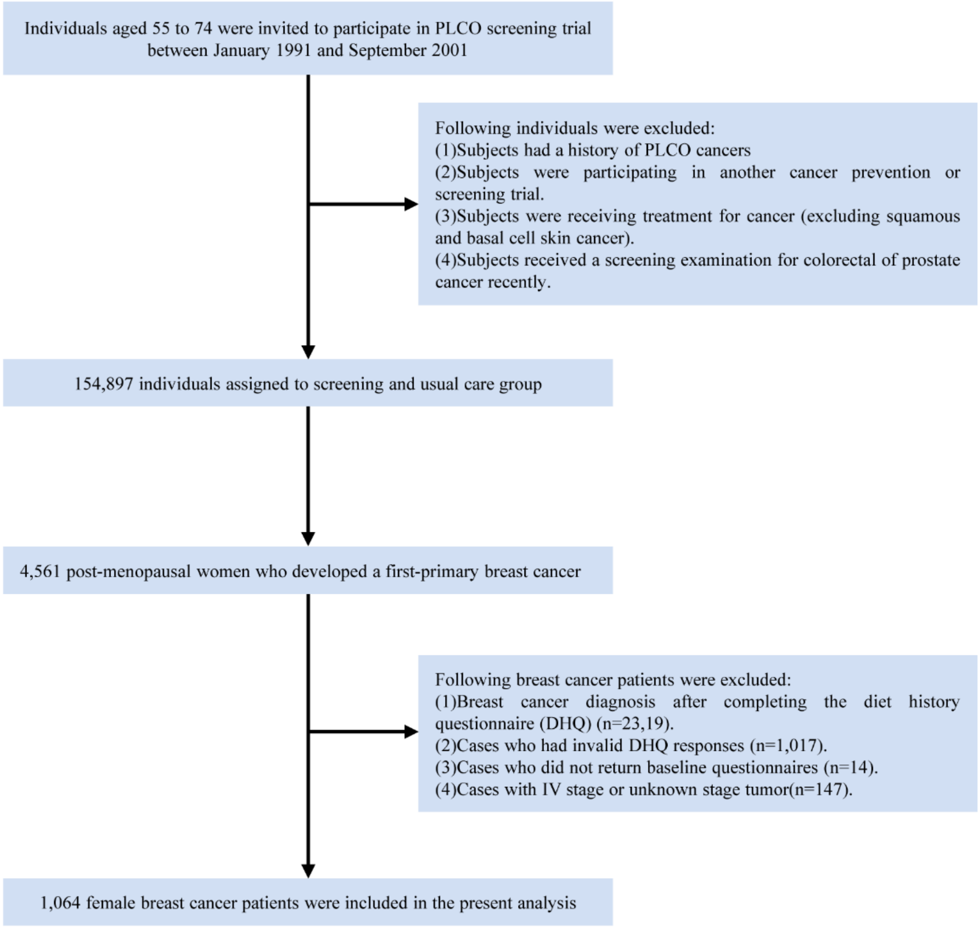

Supplementary Figure 1. The study flowchart for identifying eligible breast cancer patients. PLCO, the Prostate, Lung, Colorectal and Ovarian Cancer Screening Trial; DHQ, diet history questionnaire.

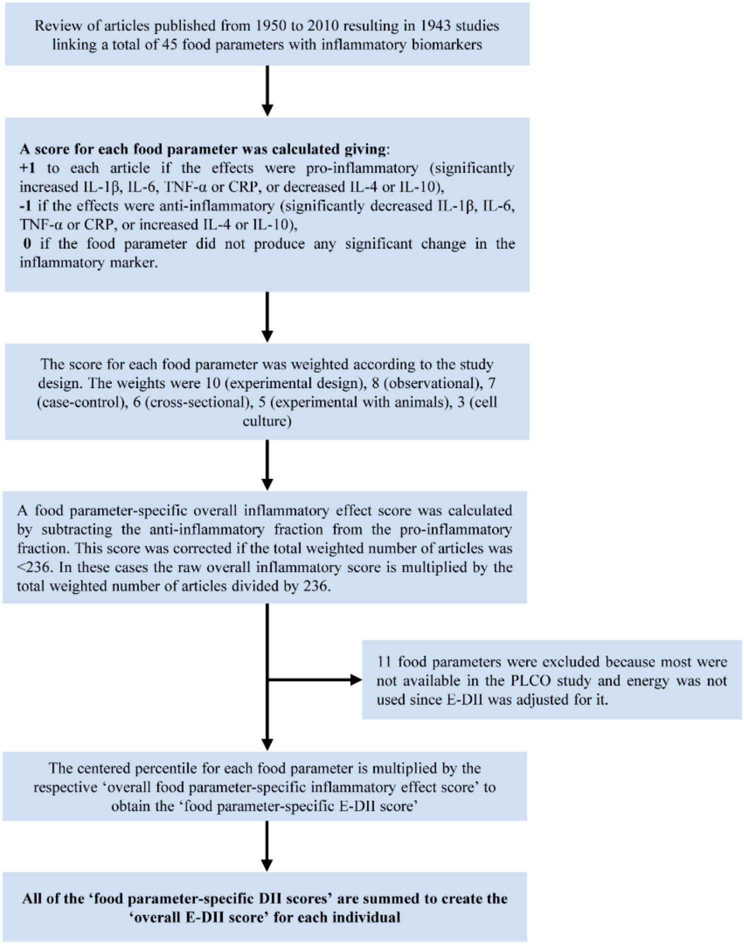

Supplementary Figure 2. Sequence of steps in creating the dietary inflammatory index (DII) in the PLCO study.

PLCO, the Prostate, Lung, Colorectal and Ovarian Cancer Screening Trial.
