# Supplementary material for: Long-term anti-inflammatory diet in relation to improved breast cancer prognosis: a prospective cohort study
Source: NPJ Breast Cancer. 2020 Aug 13;6:36. doi: 10.1038/s41523-020-00179-4 (PMC7426822; doi:10.1038/s41523-020-00179-4)
Supplement: Supplementary file 2 — Reporting Summary [file 41523_2020_179_MOESM2_ESM.pdf]

nature research

Corresponding author(s): NPJBCANCER-00630R1

Last updated by author(s): Jun 25, 2020

Reporting Summary

Nature Research wishes to improve the reproducibility of the work that we publish. This form provides structure for consistency and transparency in reporting. For further information on Nature Research policies, see our Editorial Policies and the Editorial Policy Checklist.

Statistics

For all statistical analyses, confirm that the following items are present in the figure legend, table legend, main text, or Methods section.

n/a	Confirmed	
☐	☒	The exact sample size (*n*) for each experimental group/condition, given as a discrete number and unit of measurement
☒	☐	A statement on whether measurements were taken from distinct samples or whether the same sample was measured repeatedly
☐	☒	The statistical test(s) used AND whether they are one- or two-sided<br>*Only common tests should be described solely by name; describe more complex techniques in the Methods section.*
☐	☒	A description of all covariates tested
☐	☒	A description of any assumptions or corrections, such as tests of normality and adjustment for multiple comparisons
☐	☒	A full description of the statistical parameters including central tendency (e.g. means) or other basic estimates (e.g. regression coefficient) AND variation (e.g. standard deviation) or associated estimates of uncertainty (e.g. confidence intervals)
☐	☒	For null hypothesis testing, the test statistic (e.g. *F*, *t*, *r*) with confidence intervals, effect sizes, degrees of freedom and *P* value noted<br>*Give P values as exact values whenever suitable.*
☒	☐	For Bayesian analysis, information on the choice of priors and Markov chain Monte Carlo settings
☒	☐	For hierarchical and complex designs, identification of the appropriate level for tests and full reporting of outcomes
☐	☒	Estimates of effect sizes (e.g. Cohen's *d*, Pearson's *r*), indicating how they were calculated

Our web collection on statistics for biologists contains articles on many of the points above.

Software and code

Policy information about availability of computer code

Data collection	NA
Data analysis	NA

For manuscripts utilizing custom algorithms or software that are central to the research but not yet described in published literature, software must be made available to editors and reviewers. We strongly encourage code deposition in a community repository (e.g. GitHub). See the Nature Research guidelines for submitting code & software for further information.

Data

Policy information about availability of data

All manuscripts must include a data availability statement. This statement should provide the following information, where applicable:

- Accession codes, unique identifiers, or web links for publicly available datasets
- A list of figures that have associated raw data
- A description of any restrictions on data availability

Clinical and dietary data that support the findings of this study have been deposited in PLCO trial (https://biometry.nci.nih.gov/cdas/plco/), and PLCO has the following five ClinicalTrials.gov registration numbers: NCT00002540 (Prostate), NCT01696968 (Lung), NCT01696981 (Colorectal), NCT01696994 (Ovarian), and NCT00339495 (EEMS).

Field-specific reporting

Please select the one below that is the best fit for your research. If you are not sure, read the appropriate sections before making your selection.

☒ Life sciences ☐ Behavioural & social sciences ☐ Ecological, evolutionary & environmental sciences

For a reference copy of the document with all sections, see nature.com/documents/nr-reporting-summary-flat.pdf

Life sciences study design

All studies must disclose on these points even when the disclosure is negative.

Sample size	NA
Data exclusions	To extract non-metastatic breast cancer patients who had valid DHQ responses after breast cancer diagnosis, our analytical cohort initially identified 4,561 post-menopausal women who developed a first-primary breast cancer (International Classification of Diseases for Oncology, Third Edition, codes C50.0-C50.6, C508-C509) (Supplementary Figur1). According to the exclusion criteria, we removed from consideration 2,319 women whose dietary information were collected prior to their breast cancer diagnosis; 1017 cases with invalid DHQ responses (i.e., valid DHQ responses were defined as having DHQ completion date; alive at DHQ completion; <8 missing DHQ responses; and plausible caloric intake defined as within the sex-specific first and last percentiles of total energy); 14 cases who did not return baseline questionnaires and 147 cases with IV stage or unknown stage tumor. After these exclusions, the analytical cohort included 1,064 breast cancer patients with follow-up data.
Replication	NA
Randomization	NA
Blinding	NA

Reporting for specific materials, systems and methods

We require information from authors about some types of materials, experimental systems and methods used in many studies. Here, indicate whether each material, system or method listed is relevant to your study. If you are not sure if a list item applies to your research, read the appropriate section before selecting a response.

Materials & experimental systems

n/a	Involved in the study	
☒	☐	Antibodies
☒	☐	Eukaryotic cell lines
☒	☐	Palaeontology and archaeology
☒	☐	Animals and other organisms
☒	☐	Human research participants
☐	☒	Clinical data
☒	☐	Dual use research of concern

Methods

n/a	Involved in the study	
☒	☐	ChIP-seq
☒	☐	Flow cytometry
☒	☐	MRI-based neuroimaging

Clinical data

Policy information about clinical studies

All manuscripts should comply with the ICMJE guidelines for publication of clinical research and a completed CONSORT checklist must be included with all submissions.

Clinical trial registration	NCT00002540 (Prostate), NCT01696968 (Lung), NCT01696981 (Colorectal), NCT01696994 (Ovarian), and NCT00339495 (EEMS)
Study protocol	Clinical and dietary data that support the findings of this study have been deposited in PLCO trial (https://biometry.nci.nih.gov/cdas/plco/)
Data collection	PLCO was sponsored by the National Cancer Institute (NCI), conducted in the United States, and its purpose was to determine the effects of screening on cancer-related mortality and secondary endpoints29. In total, 154,897 eligible participants (76,682 males and 78,215 females), aged 55-74 years were enrolled from November 1993 to July 2001. Participants were individually randomized to the intervention group or the control group in equal proportions. Data collection on cancer diagnoses and deaths from all causes that occurred through July 31, 2011.
Outcomes	cancer incidence
